# Ruptured focal nodular hyperplasia observed during follow-up: a case report

**DOI:** 10.1186/s40792-017-0320-4

**Published:** 2017-03-17

**Authors:** Masahiko Kinoshita, Shigekazu Takemura, Shogo Tanaka, Genya Hamano, Tokuji Ito, Takanori Aota, Masaki Koda, Masahiko Ohsawa, Shoji Kubo

**Affiliations:** 1 Department of Hepato-Biliary-Pancreatic Surgery, Osaka City University Graduate School of Medicine, 1-4-3 Asahimachi, Abenoku, Osaka, 545-8585 Japan; 2 Department of Diagnostic Pathology, Osaka City University Graduate School of Medicine, 1-4-3 Asahimachi, Abenoku, Osaka, 545-8585 Japan

**Keywords:** Focal nodular hyperplasia, Rupture, Hemorrhage

## Abstract

**Background:**

Focal nodular hyperplasia (FNH) is the second most common benign hepatic tumor and is very rarely complicated by hemorrhage or rupture. Although thought to be extremely rare, there have been several reports of hemorrhage caused by ruptured FNH. Herein, we report the case of a patient with ruptured FNH, who subsequently developed hemorrhage during follow-up.

**Case presentation:**

A 32-year-old man was admitted to our department for an asymptomatic hepatic tumor in segments 4 and 5 (S4/5), which measured 8 cm in diameter and observed to project from the liver. Imaging and pathologic examination of a biopsy specimen confirmed the diagnosis of FNH. Three years after the diagnosis, the patient was readmitted to our hospital because of sudden onset of upper abdominal pain. Dynamic abdominal computed tomography revealed ascites around the tumor with high-density areas that were considered to represent hematoma caused by ruptured FNH. Transcatheter arterial embolization (TAE) was performed to stop the hemorrhage. One month after TAE, S4/5 of the liver was resected; macroscopic findings revealed that a large part of the tumor was composed of necrotic tissue and hematoma. Pathological examination using hematoxylin–eosin staining and immunohistochemical examination indicated a final diagnosis of FNH rupture and hemorrhage.

**Conclusion:**

Although a well-established diagnosis of FNH usually requires no treatment or surveillance, careful examination remains necessary when the FNH is large and projects from the liver because of the possibility of rupture and hemorrhage.

## Background

Focal nodular hyperplasia (FNH) is the second most common benign hepatic tumor after cavernous hemangioma [1–3]. Although a large FNH is often associated with significant symptoms, almost all tumors remain stable in size and do not develop complications, such as hemorrhage and rupture. Therefore, FNH usually requires no treatment or surveillance if the diagnosis is well established [4, 5]. However, hemorrhage caused by ruptured FNH may develop [5–13], although such instances appear to be extremely rare. Here, we reported the case of a patient with ruptured FNH that caused hemorrhage during follow-up.

## Case presentation

**Fig. 1:**
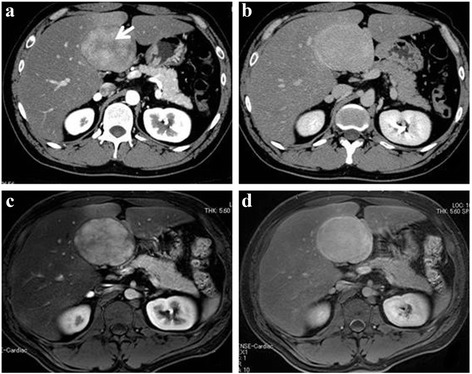
Diagnostic imaging findings upon first admission. Dynamic abdominal CT imaging reveals an 8-cm mass projecting from the liver. Contrast enhancement is mainly on the border, and it is observed in the early phase (**a**) until the late phase (**b**). In the early phase, a low-density area in the tumor is considered as a central stellate scar (*arrow*). Dynamic abdominal MRI shows contrast enhancement in the early phase (**c**) until the hepatobiliary phase (**d**)

**Fig. 2:**
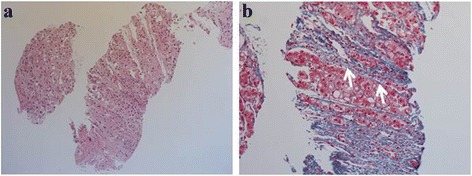
Pathologic examination of a percutaneous liver biopsy sample. Fibrous connective tissue, including a bile ductule (**b**, *arrow*), and absence of a normal portal vein in the specimen are shown. **a** Hematoxylin–eosin staining (×100) and **b** Azan staining (×200)

A 32-year-old man was admitted to our department because of a hepatic tumor. Although the patient was asymptomatic, his previous medical history included chronic alcohol consumption for the past 8 years. Dynamic abdominal computed tomography (CT) scan revealed an 8-cm mass that projected from segments 4–5 (S4/5) of the liver. The tumor exhibited contrast enhancement, mainly on the border, from the early phase until the late phase. The tumor contained a low-density area that was considered as a central stellate scar (Fig. 1a, b). Dynamic abdominal magnetic resonance imaging (MRI) showed a mass with contrast enhancement during the early phase until the hepatobiliary phase (Fig. 1c, d). Pathologic examination of a percutaneous biopsy sample from the tumor showed fibrous connective tissue and a bile ductule without a normal portal vein (Fig. 2a, b). The tumor was diagnosed as FNH. The patient was advised regular follow-up with no treatment.

**Fig. 3:**
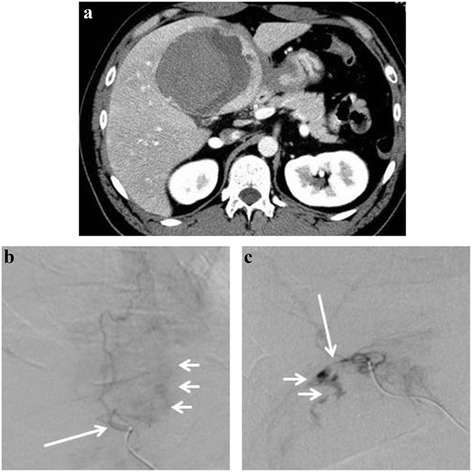
Diagnostic imaging findings at 3 years after initial admission. Dynamic abdominal CT shows minimal ascites around the tumor. Most of the tumor is depicted as a low-density area with some high-density areas that indicate hematoma formation (**a**). Emergency arterial angiography is performed (**b**, **c**). The tumor is stained (**b**, *short arrow*) from the arterial branches of S4 (**b**, *long arrow*) and S5 (**c**, *arrow*). Extravasation (**c**, *short arrow*) from the arterial branch of S5 is observed, and TAE was performed

Three years after diagnosis, he was readmitted to our hospital because of sudden onset of upper abdominal pain without any preceding traumatic events. His hemodynamic status on admission was nearly stable, except for tachycardia (117 beats per minute). Further physical examination revealed a palpable induration with tenderness and peritoneal signs on the epigastrium. Laboratory test results on admission revealed no anemia (hemoglobin, 15.7 g/dl, and hematocrit, 45.2%) and an increased white blood cell count (17,700 cells/μl). The serum levels of C-reactive protein (30.2 mg/dl), aspartate aminotransferase (256 U/l), and alanine aminotransferase (491 U/l) were also elevated. Dynamic abdominal CT scan showed a small amount of ascites around the tumor. Most of the tumor was observed as a low-density area, but there were high-density areas, which were considered as hematoma around the tumor (Fig. 3a). These findings indicated that the abdominal pain was due to a ruptured FNH.

Although extravasation was not obvious on dynamic CT and the hemodynamic status of the patient was almost stable, the possibility of continuous hemorrhage could not be ruled out because of the presence of hematoma around the tumor, severe abdominal pain, and tachycardia. Although the tumor had been initially diagnosed as FNH, we deemed that hepatic angiography was necessary for reassessment and accurate diagnosis of the hepatic lesion because of the extreme rarity of a ruptured FNH. Hepatic angiography showed patent arterial branches of the S4 and S5 without a spoke-wheel pattern, but with extravasation.

**Fig. 4:**
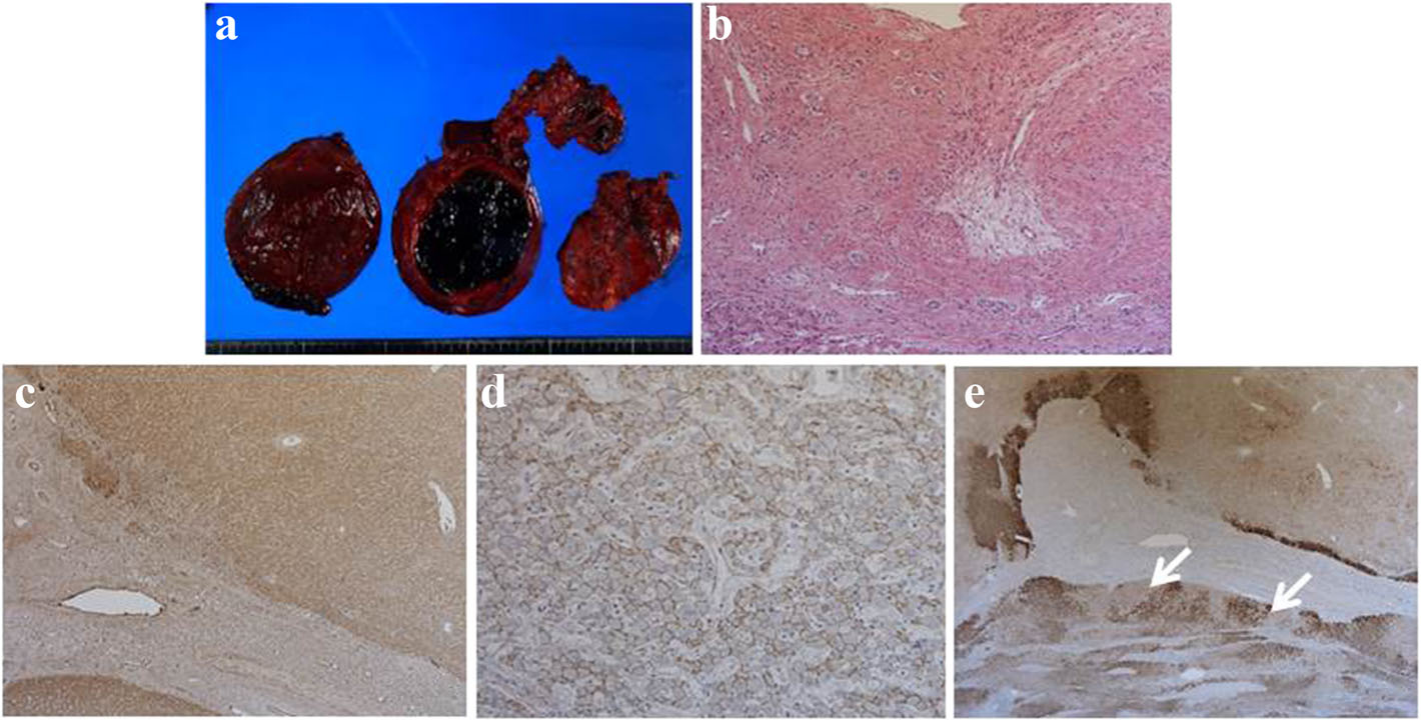
Macroscopic and histopathologic findings of the surgical specimen. Grossly, most of the tumorous tissue is replaced by hemorrhage (**a**). Hematoxylin–eosin staining shows the absence of atypical cells and normal portal vein area, but there are hepatocyte proliferation and expanded blood vessels (**b**, ×40). Immunohistochemical analyses (**c**–**e**) show inactivation of β-catenin and no attenuation of the LFABP (**c**, β-catenin, ×40, and **d**, LFABP, ×40). Glutamine synthetase in the tumor region presents as the so-called geographic map-like pattern (**e**, *arrows* ×40). *LFABP*, liver fatty acid-binding protein

Transcatheter arterial embolization (TAE) of the arterial branches of S4 and S5 was performed to stop the bleeding (Fig. 3b, c). One month after TAE, his condition improved by conservative treatment and elective surgery was performed. On laparotomy, a large S4/5 hepatic tumor adherent to the omentum, minimal ascites, and an old hematocele were observed. Partial resection of the S4/5 was performed. Macroscopic findings of the surgical specimen showed that most of the tumor was composed of necrotic tissue and hematoma (Fig. 4a). Pathologic examination of the hematoxylin–eosin-stained biopsy sample showed no atypical cells, hepatocyte proliferation, and expanded blood vessels. An area with normal portal vein was not observed (Fig. 4b). Immunohistochemical analyses revealed inactivation of β-catenin and no attenuation of liver fatty acid-binding protein (Fig. 4c, d). The expression of glutamine synthetase (GS) in the tumor region demonstrated the so-called “map-like” pattern (Fig. 4e). From these findings, a final diagnosis of ruptured FNH was made. Postoperatively, the patient was stable and has survived symptom free for 2 years.

### Discussion

FNH accounts for approximately 8% of all primary hepatic tumors, with an estimated prevalence of 0.9% in the general population [2]. FNH occurs in all age groups in both men and women, but it is more frequent in women aged 20–50 years (i.e., in the reproductive age) [14], especially in those using oral contraceptives [15, 16]. In fact, an association between female hormones, such as estrogen or progesterone, and the occurrence and increasing size of FNH have been suspected; however, this remains to be established [17, 18]. Although the etiology of FNH remains undefined, it may represent a tissue-specific ischemic response to congenital vascular anomalies [19]. Alternatively, this lesion may result from an arteriovenous malformation exerting abnormal pressure on the surrounding sinusoids and portal vein branches [20]. An anomalous arterial supply in FNH has been demonstrated by arteriography [21].

Most FNHs are incidentally discovered with few clinical clues [3]. Only 20% of patients reported signs and symptoms secondary to a liver mass [1, 16, 21, 22]. The presence of a central stellate scar in the tumor is a characteristic finding on abdominal ultrasonography, CT, and MRI [14, 23]. Compared with abdominal gray-scale ultrasonography or CT, MRI has a high diagnostic yield for FNH, with sensitivity of 68–70% and specificity of 98–100% [14, 24, 25]. Particularly, MRI with hepatobiliary-specific contrast agents, such as gadoxetic acid and gadobenate dimeglumine, is very useful in diagnosing FNH, and it has been reported to have 96–97% specificity and 96% positive predictive value [25, 26]. In the hepatobiliary phase of gadoxetate disodium-enhanced MRI (Gd-EOB-MRI), FNH usually appears as an iso- or hyperintense lesion [27], compared with malignant tumors and hepatocellular adenoma (HCA), majority of which demonstrate hypointensity [28]. Similarly, contrast-enhanced ultrasonography (CEUS) can contribute to accurate diagnosis of FNH by real-time evaluation of vascularization and detection of the characteristic spoke-wheel pattern. FNH can present on CEUS as a hyper- or isointense lesion in the Kupffer phase because of the presence of Kupffer cells; in addition, CEUS can differentiate between benign and malignant focal hepatic lesions [29–31]. In this present case, tumor was recognized in the hepatobiliary phase of Gd-EOB-MRI as a hyperintense mass that contained a low-density area, which was a central stellate scar. However, we assessed that an accurate diagnosis by pathologic examination of a liver biopsy specimen should be performed because of some atypical features, such as male gender and an equivocal finding of a central scar. Although CEUS was not performed in our present case, evaluation of real-time dynamism and the Kupffer phase on CEUS should be considered for non-invasive diagnosis of FNH before biopsy.

Microscopically, FNH is a confluent mass of several small nodules of benign-appearing hepatocytes, partially or surrounded by fine bands of fibrous tissue containing proliferating bile ductules and vessels. It lacks a capsule and grows in an expansive manner to compress the adjacent hepatic parenchyma, which may exhibit a pattern of sinusoidal congestion [32–34]. The value of immunohistochemistry in diagnosis of FNH has been recently explored. GS is an enzyme involved in detoxification of ammonia by combining it with glutamate to produce the amino acid glutamine. In the normal liver, GS expression is limited to a narrow rim of hepatocytes around the central vein [35, 36]. This zonation is thought to be the result of β-catenin activation in centrizonal hepatocytes, which may be the result of Wnt signaling from the central vein [35, 36]. In FNH, there is expansion of the β-catenin-activated centrizonal region, which leads to overexpression in a characteristic “geographic map-like” pattern [37, 38], as in the present case. A small subset of β-catenin-activated HCAs may present with diffuse cytoplasmic expression of GS, but the map-like pattern typical of FNH is not observed. GS can provide strong evidence to differentiate between FNH and β-catenin-activated HCA [38, 39].

Several studies reported that FNH does not have an aggressive or malignant course [4, 19, 22]. Therefore, FNH usually requires no treatment or surveillance if the diagnosis is well established. However, although extremely rare, FNH associated with intraperitoneal hemorrhage has been reported in ten cases, including the present case, based on review of English literature since 1974 (Table 1) [5–13]. All cases, except this patient, were women; fatal hemorrhage occurred in one patient in late pregnancy [13]. The maximum tumor diameter ranged from 1 to 10 cm (median, 7 cm). Among the cases in which the location could be estimated, the tumors were located at or near the liver surface. Li et al. recommended surgical resection over observation for large FNH (>5 cm), irrespective of the presence of symptoms, because surgery was associated with less mortality and it can estimate the possibility of rupture or hemorrhage [12]. Surgical resection was performed in nine patients because one patient was diagnosed during autopsy [13]; in three patients, including this patient, preoperative TAE was performed.

**Table 1:** Documented patients of hemorrhage caused by FNH

Patients	Author	Publication year	Age (years)	Gender (M/F)	Maximum diameter of tumor (cm)	Number of tumor	Location	Treatment	Preoperative TAE
1	Mays ET	1974	26	F	10	1	Anterior segment	Surgery	No
2	Becker YT	1995	18	F	4.5	2	Anterior and medial segment	Surgery	No
3	Hardwigsen J	2001	37	F	5	1	Segment 7	Surgery	Yes
4	Bathe OF	2003	27	F	6	1	Right lobe	Surgery	No
5	Rahili A	2005	35	F	9.8	1	Lobus caudatus	Surgery	No
6	Chang SK	2005	42	F	10	1	Segment 7/8	Surgery	No
7	Demarco MP	2006	37	F	5.2	4	Lateral segment (2 tumors), segment 4B, and posterior segment (hemorrhagic lesion)	Surgery	No
8	Li T	2006	26	F	15	ND	Left robe	Surgery	Yes
9	Yajima D	2013	23	F	1	1	Right lobe	Revealed at autopsy	
10	Kinoshita M	2016	35	M	8	1	Segment 4A/5	Surgery	Yes

*M* male, *F* female, *ND* not described in the report, *TAE* transcatheter arterial embolization

Spontaneous rupture occurs in 3–26% of hepatocellular carcinoma (HCC) cases, and it is regarded as a life-threatening condition [40–45]. Some studies reported that TAE is effective in controlling bleeding from a ruptured HCC in the acute phase [46–48]. Hsueh et al. reported that the prognosis of a ruptured HCC after TAE was better than that after conservative treatment and that TAE achieved successful hemostasis in 99% of patients [49]. For patients with ruptured FNH, TAE may be also effective in controlling bleeding in the acute phase. In the present case, extravasation was revealed on hepatic angiography and TAE was performed. Staged resection after TAE should be considered for selected patients based on the presentation of repeated hemorrhage and extensive tumor necrosis, as observed in the present case. TAE and staged resection should be considered a standard treatment for ruptured FNH.

## Conclusions

In conclusion, although majority of FNH cases are asymptomatic and require no treatment, spontaneous rupture may occur in some patients during the follow-up period. Although ruptured FNH is extremely rare, careful examination is necessary for a large FNH that projects from the liver because of the risk of spontaneous rupture, which can present as sudden onset of abdominal pain.

## Abbreviations

CEUS: Contrast-enhanced ultrasonography
CT: Computed tomography
FNH: Focal nodular hyperplasia
Gd-EOB-MRI: Gadoxetate disodium-enhanced MRI
GS: Glutamine synthetase
HCA: Hepatocellular adenoma
HCC: Hepatocellular carcinoma
LFABP: Liver fatty acid-binding protein
MRI: Magnetic resonance imaging
TAE: Transcatheter arterial embolization

## References

[1] Ishak KG, Rabin L (1975). Benign tumors of the liver. Med Clin North Am.

[2] Vilgrain V (2006). Focal nodular hyperplasia. Eur J Radiol.

[3] Kerlin P, Davis GL, McGill DB, Weiland LH, Adson MA, Sheedy PF (1983). Hepatic adenoma and focal nodular hyperplasia: clinical, pathologic, and radiologic features. Gastroenterology.

[4] Adson MA (1986). Mass lesions of the liver. Mayo Clin Proc.

[5] Becker YT, Raiford DS, Webb L, Wright JK, Chapman WC, Pinson CW (1995). Rupture and hemorrhage of hepatic focal nodular hyperplasia. Am Surg.

[6] Mays ET, Christopherson WM, Barrows GH (1974). Focal nodular hyperplasia of the liver. Possible relationship to oral contraceptives. Am J Clin Pathol.

[7] Hardwigsen J, Pons J, Veit V, Garcia S, Le Treut YP (2001). A life-threatening complication of focal nodular hyperplasia. J Hepatol.

[8] Bathe OF, Mies C, Franceschi D, Casillas J, Livingstone AS (2003). Massive hemorrhage and infarction complicating focal nodular hyperplasia of the liver. HPB.

[9] Chang SK, Chung YF, Thng CH, Loo HW (2005). Focal nodular hyperplasia presenting as acute abdomen. Singapore Med J.

[10] Rahili A, Cai J, Trastour C, Juwid A, Benchimol D, Zheng M (2005). Spontaneous rupture and hemorrhage of hepatic focal nodular hyperplasia in lobus caudatus. J Hepatobiliary Pancreat Surg.

[11] Demarco MP, Shen P, Bradley RF, Levine EA (2006). Intraperitoneal hemorrhage in a patient with hepatic focal nodular hyperplasia. Am Surg.

[12] Li T, Qin LX, Ji Y, Sun HC, Ye QH, Wang L (2007). Atypical hepatic focal nodular hyperplasia presenting as acute abdomen and misdiagnosed as hepatocellular carcinoma. Hepatol Res.

[13] Yajima D, Kondo F, Nakatani Y, Saitoh H, Hayakawa M, Sato Y (2013). A fatal case of subcapsular liver hemorrhage in late pregnancy: a review of hemorrhages caused by hepatocellular hyperplastic nodules. J Forensic Sci.

[14] Assy N, Nasser G, Djibre A, Beniashvili Z, Elias S, Zidan J (2009). Characteristics of common solid liver lesions and recommendations for diagnostic workup. World J Gastroenterol.

[15] Herman P, Pugliese V, Machado MA, Montagnini AL, Salem MZ, Bacchella T (2000). Hepatic adenoma and focal nodular hyperplasia: differential diagnosis and treatment. World J Surg.

[16] Pain JA, Gimson AE, Williams R, Howard ER (1991). Focal nodular hyperplasia of the liver: results of treatment and options in management. Gut.

[17] Kapp N, Curtis KM (2009). Hormonal contraceptive use among women with liver tumors: a systematic review. Contraception.

[18] Mathieu D, Kobeiter H, Maison P, Rahmouni A, Cherqui D, Zafrani ES (2000). Oral contraceptive use and focal nodular hyperplasia of the liver. Gastroenterology.

[19] Knowles DM, Casarella WJ, Johnson PM, Wolff M (1978). The clinical, radiologic, and pathologic characterization of benign hepatic neoplasms. Alleged association with oral contraceptives. Medicine.

[20] Iwatsuki S, Todo S, Starzl TE (1990). Excisional therapy for benign hepatic lesions. Surg Gynecol Obstet.

[21] Whelan TJ, Baugh JH, Chandor S (1973). Focal nodular hyperplasia of the liver. Ann Surg.

[22] Malt RA (1985). Surgery for hepatic neoplasms. N Engl J Med.

[23] Grazioli L, Morana G, Federle MP, Brancatelli G, Testoni M, Kirchin MA (2001). Focal nodular hyperplasia: morphologic and functional information from MR imaging with gadobenate dimeglumine. Radiology.

[24] Hussain SM, Terkivatan T, Zondervan PE, Lanjouw E, de Rave S, Ijzermans JN (2004). Focal nodular hyperplasia: findings at state-of-the-art MR imaging, US, CT, and pathologic analysis. Radiographics.

[25] Bieze M, van den Esschert JW, Nio CY, Verheij J, Reitsma JB, Terpstra V (2012). Diagnostic accuracy of MRI in differentiating hepatocellular adenoma from focal nodular hyperplasia: prospective study of the additional value of gadoxetate disodium. AJR Am J Roentgenol.

[26] Fowler KJ, Brown JJ, Narra VR (2011). Magnetic resonance imaging of focal liver lesions: approach to imaging diagnosis. Hepatology.

[27] Zech CJ, Grazioli L, Breuer J, Reiser MF, Schoenberg SO (2008). Diagnostic performance and description of morphological features of focal nodular hyperplasia in Gd-EOB-DTPA-enhanced liver magnetic resonance imaging: results of a multicenter trial. Investig Radiol.

[28] Giovanoli O, Heim M, Terracciano L, Bongartz G, Ledermann HP (2008). MRI of hepatic adenomatosis: initial observations with gadoxetic acid contrast agent in three patients. AJR Am J Roentgenol.

[29] Dietrich CF, Schuessler G, Trojan J, Fellbaum C, Ignee A (2005). Differentiation of focal nodular hyperplasia and hepatocellular adenoma by contrast-enhanced ultrasound. Br J Radiol.

[30] Tanaka S, Hamada Y, Ioka T, Sugiyama T, Akamatsu I, Takakura R (2005). Contrast-enhanced multiphase dynamic ultrasonography for the characterization of liver tumors. J Med Ultrason.

[31] Inoue T, Hyodo T, Korenaga K, Murakami T, Imai Y, Higaki A (2016). Kupffer phase image of Sonazoid-enhanced US is useful in predicting a hypervascularization of non-hypervascular hypointense hepatic lesions detected on Gd-EOB-DTPA-enhanced MRI: a multicenter retrospective study. J Gastroenterol.

[32] Wanless IR, Mawdsley C, Adams R (1985). On the pathogenesis of focal nodular hyperplasia of the liver. Hepatology.

[33] Gold JH, Guzman IJ, Rosai J (1978). Benign tumors of the liver. Pathologic examination of 45 cases. Am J Clin Pathol.

[34] Knowles DM, Wolff M (1976). Focal nodular hyperplasia of the liver: a clinicopathologic study and review of the literature. Hum Pathol.

[35] Walther Z, Jain D (2011). Molecular pathology of hepatic neoplasms: classification and clinical significance. Pathol Res Int.

[36] Joseph NM, Ferrell LD, Jain D, Torbenson MS, Wu TT, Yeh MM (2014). Diagnostic utility and limitations of glutamine synthetase and serum amyloid-associated protein immunohistochemistry in the distinction of focal nodular hyperplasia and inflammatory hepatocellular adenoma. Mod Pathol.

[37] Bioulac-Sage P, Balabaud C, Zucman-Rossi J (2010). Focal nodular hyperplasia, hepatocellular adenomas: past, present, future. Gastroenterol Clin Biol.

[38] Bioulac-Sage P, Laumonier H, Rullier A, Cubel G, Laurent C, Zucman-Rossi J (2009). Over-expression of glutamine synthetase in focal nodular hyperplasia: a novel easy diagnostic tool in surgical pathology. Liver Int.

[39] Shafizadeh N, Genrich G, Ferrell L, Kakar S (2014). Hepatocellular adenomas in a large community population, 2000 to 2010: reclassification per current World Health Organization classification and results of long-term follow-up. Hum Pathol.

[40] Chen MF, Hwang TL, Jeng LB, Jan YY, Wang CS (1995). Clinical experience with hepatic resection for ruptured hepatocellular carcinoma. Hepatogastroenterology.

[41] Vergara V, Muratore A, Bouzari H, Polastri R, Ferrero A, Galatola G (2000). Spontaneous rupture of hepatocellular carcinoma: surgical resection and long-term survival. Eur J Surg Oncol.

[42] Liu CL, Fan ST, Lo CM, Tso WK, Poon RT, Lam CM (2001). Management of spontaneous rupture of hepatocellular carcinoma: single-center experience. J Clin Oncol.

[43] Bassi N, Caratozzolo E, Bonariol L, Ruffolo C, Bridda A, Padoan L (2010). Management of ruptured hepatocellular carcinoma: implications for therapy. World J Gastroenterol.

[44] Chen CY, Lin XZ, Shin JS, Lin CY, Leow TC, Chen CY (1995). Spontaneous rupture of hepatocellular carcinoma. A review of 141 Taiwanese cases and comparison with nonrupture cases. J Clin Gastroenterol.

[45] Tanaka S, Kaibori M, Ueno M, Wada H, Hirokawa F, Nakai T, et al. Surgical outcomes for the ruptured hepatocellular carcinoma: multicenter analysis with a case-controlled study. J Gastrointest Surg. 2016;20:2021-34. 10.1007/s11605-016-3280-2 27718151

[46] Lai EC, Lau WY (2006). Spontaneous rupture of hepatocellular carcinoma: a systematic review. Arch Surg.

[47] Kung CT, Liu BM, Ng SH, Lee TY, Cheng YF, Chen MC (2008). Transcatheter arterial embolization in the emergency department for hemodynamic instability due to ruptured hepatocellular carcinoma: analysis of 167 cases. AJR Am J Roentgenol.

[48] Li AJ, Zhou WP, Wu MC, Luo XJ (2007). Hepatectomy after primary repair of ruptured liver cancer. Hepatobiliary Pancreat Dis Int.

[49] Hsueh KC, Fan HL, Chen TW, Chan DC, Yu JC, Tsou SS (2012). Management of spontaneously ruptured hepatocellular carcinoma and hemoperitoneum manifested as acute abdomen in the emergency room. World J Surg.

